# Indirect-Neural-Approximation-Based Fault-Tolerant Integrated Attitude and Position Control of Spacecraft Proximity Operations

**DOI:** 10.3390/s22051726

**Published:** 2022-02-23

**Authors:** Fawaz W. Alsaade, Qijia Yao, Mohammed S. Al-zahrani, Ali S. Alzahrani, Hadi Jahanshahi

**Affiliations:** 1 Department of Computer Science, College of Computer Sciences and Information Technology, King Faisal University, Al-Ahsa 31982, Saudi Arabia; falsaade@kfu.edu.sa; 2 School of Aerospace Engineering, Beijing Institute of Technology, Beijing 100081, China; 3 Department of Computer Networks and Communications, College of Computer Sciences and Information Technology, King Faisal University, Al-Ahsa 31982, Saudi Arabia; malzahrani@kfu.edu.sa; 4 Department of Computer Engineering, College of Computer Sciences and Information Technology, King Faisal University, Al-Ahsa 31982, Saudi Arabia; aalzahrani@kfu.edu.sa; 5 Department of Mechanical Engineering, University of Manitoba, Winnipeg, MB R3T 5V6, Canada; jahanshahi.hadi90@gmail.com

**Keywords:** neural adaptive control, fault-tolerant control, integrated attitude and position control, spacecraft proximity operations, indirect neural approximation, Lyapunov analysis

## Abstract

In this paper, a neural adaptive fault-tolerant control scheme is proposed for the integrated attitude and position control of spacecraft proximity operations in the presence of unknown parameters, disturbances, and actuator faults. The proposed controller is made up of a relative attitude control law and a relative position control law. Both the relative attitude control law and relative position control law are designed by adopting the neural networks (NNs) to approximate the upper bound of the lumped unknowns. Benefiting from the indirect neural approximation, the proposed controller does not need any model information for feedback. In addition, only two adaptive parameters are required for the indirect neural approximation, and the online calculation burden of the proposed controller is therefore significantly reduced. Lyapunov analysis shows that the overall closed-loop system is ultimately uniformly bounded. The proposed controller can ensure the relative attitude, angular velocity, position, and velocity stabilize into the small neighborhoods around the origin. Lastly, the effectiveness and superior performance of the proposed control scheme are confirmed by a simulated example.

## 1. Introduction

Nowadays, with the rapid development of sensing and control technologies, space missions have become increasingly complicated. The spacecraft proximity operation plays an important role in various space missions, such as rendezvous and docking, active debris removal, and on-orbit servicing. The relative attitude and position control is a critical technique for spacecraft proximity operations. During the proximity operations, the chaser and target are inevitably affected by uncertain parameters and disturbances. Even worse, the parameters of the target may be fully unknown for noncooperative proximity operations. In addition, the chaser also frequently suffers from actuator faults due to the harsh space environment. The presence of unknown parameters, disturbances, and actuator faults bring great difficulty to the relative attitude and position control of spacecraft proximity operations. Traditionally, the spacecraft relative attitude and position control systems are often designed independently. However, the inherent couplings between the relative attitude and position are neglected in this way and these controllers cannot be directly applied to the spacecraft proximity operations, especially when high control accuracy is required. The integrated attitude and position control based on the six-degree-of-freedom (6-DOF) dynamic model of spacecraft proximity operations is an effective solution to this problem.

Until recently, many relevant results have been reported for the integrated attitude and position control of spacecraft proximity operations. Singla et al. [1] designed a model reference adaptive output feedback control law for the spacecraft rendezvous and docking under measurement uncertainties. Kristiansen et al. [2] presented three nonlinear control solutions for the 6-DOF spacecraft coordination control based on the integrator backstepping and passivity-based control, respectively. In [3,4], an integrated nonlinear optimal control approach was developed for the spacecraft proximity operations. Zhang and Duan [5] proposed a robust adaptive backstepping control scheme for the integrated translational and rotational motion of spacecraft with actuator misalignment. In [6,7], several robust optimal sliding mode control methods were carried out for the coupled attitude and position maneuvers of spacecraft. Sun and Huo [8] designed a 6-DOF integrated adaptive backstepping controller for the spacecraft proximity operations under uncertainties. In [9,10], integrated robust adaptive control approaches were developed for the relative position tracking and attitude synchronization for spacecraft rendezvous. In [11,12], disturbance observer-based robust control approaches were proposed for the spacecraft proximity and docking with input saturation. Hu et al. [13] presented a robust fault-tolerant tracking control scheme for the spacecraft proximity operations by utilizing the adaptive sliding mode control technique. Wang and Ji [14] designed two backstepping control schemes for the relative motion control of spacecraft rendezvous based on the input-to-state stable property and finite-time control technique, respectively. In [15], an adaptive nonlinear state feedback control method was proposed for the fault-tolerant constrained pose control of cooperative spacecraft rendezvous and docking. Zhou et al. [16] developed an adaptive sliding mode method for the robust attitude and position tracking of spacecraft proximity operations by integrating with an unscented Kalman filter. In [17,18,19], several adaptive nonsingular terminal sliding mode control laws were designed for the fixed-time, 6-DOF tracking control of noncooperative spacecraft fly-around missions. In addition, there have been also some research studies concerned with 6-DOF integrated controls in spacecraft based on the dual quaternion representation [20,21,22,23,24,25,26,27].

It should be noted that most of the above controllers require prior knowledge of nominal model information for feedback. Nevertheless, the physical parameters of the chaser and the target may be fully unknown in some extreme cases. The intelligent approximation is an efficient tool to construct the model-free controllers, owing to the powerful learning capability of the neural network (NN) and fuzzy logic system. By adopting the NNs or fuzzy logic systems to approximate the lumped unknowns, the intelligent control does not need any model information for feedback. In [28,29], robust adaptive backstepping NN control strategies were presented for the spacecraft rendezvous and docking with input saturation. Sun et al. [30] developed an adaptive fuzzy backstepping controller for the pose tracking of spacecraft rendezvous and proximity maneuvers under uncertainties. However, all of the above intelligent controllers involve a large number of adaptive parameters, which restricts their applications in practical engineering, especially considering the onboard computer has limited online calculation capability.

Motivated by the above discussions, this paper proposes a neural adaptive fault-tolerant control scheme for the integrated attitude and position control of spacecraft proximity operations in the presence of unknown parameters, disturbances, and actuator faults. The proposed controller is made up of a relative attitude control law and a relative position control law. In comparison with most of the existing investigations, the main contributions of this research are summarized as follows:

- Both the relative attitude control law and relative position control law are designed by integrating with the neural approximation. Benefiting from this design, the proposed controller is model-free and strongly robust against the lumped unknowns in 6-DOF dynamics;
- Rather than the conventional intelligent approximation [28,29,30], in which the NNs and fuzzy logic systems are introduced to directly approximate the lumped unknowns, the indirect neural approximation is exploited in this paper by adopting the NNs to approximate the upper bound of the lumped unknowns. In this way, only two adaptive parameters are required for the indirect neural approximation, and the online calculation burden of the proposed controller is therefore significantly reduced;
- Lyapunov analysis shows that the overall closed-loop system is ultimately uniformly bounded. The proposed controller can ensure that the relative attitude, angular velocity, position, and velocity stabilize into the small neighborhoods around the origin.

The remainder of this paper is arranged as follows: Section 2 describes the problem and gives some preliminaries. Section 3 introduces the control methodology and provides the Lyapunov analysis. Section 4 performs a simulated example. Lastly, Section 5 presents the main conclusions of this study.

## 2. Problem Statement and Preliminaries

### 2.1. The 6-DOF Dynamics of Spacecraft Proximity Operations

**Figure 1:**
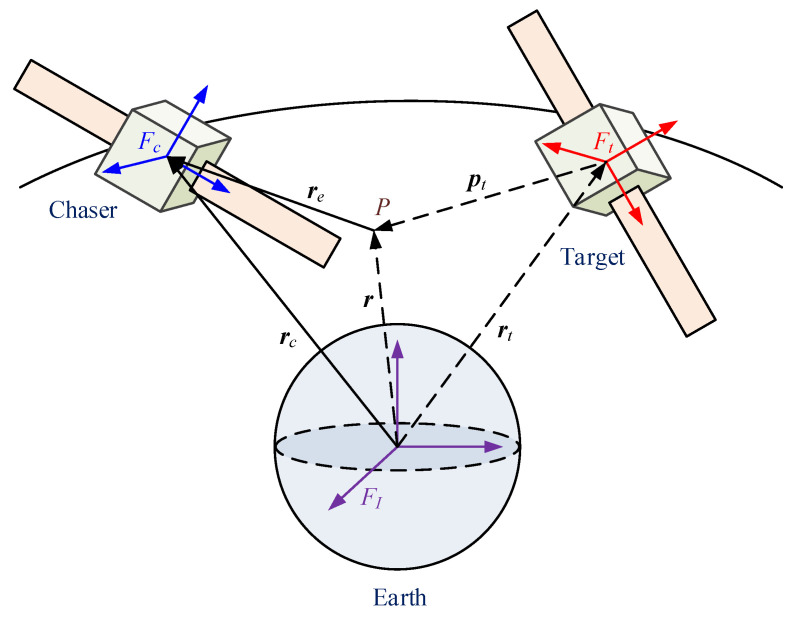
Diagram of the spacecraft proximity operation system.

Consider the spacecraft proximity operation system depicted in Figure 1, in which a chaser is approaching a freely tumbling target. P denotes the desired docking point, which is fixed with respect to the target. Three coordinate frames are introduced to describe the 6-DOF dynamics of the spacecraft proximity operation. They are the earth-centered inertial frame $F_{I}$, the chaser’s body-fixed frame $F_{c}$, and the target’s body-fixed frame $F_{t}$, respectively.

The modified Rodrigues parameters (MRPs) are utilized to represent the attitude orientation of the chaser. Then, the attitude and position dynamics of the chaser can be expressed in frame $F_{c}$ as

(1) $$\left\{ \begin{array}{l} \dot{\sigma }=G\left(\sigma \right)\omega ,\\ J\dot{\omega }+S\left(\omega \right)J\omega =\Gamma_{\tau }u_{\tau }+d_{\tau },\\ \dot{r}=v-S\left(\omega \right)r,\\ m\dot{v}+mS\left(\omega \right)v=\Gamma_{f}u_{f}+d_{f}, \end{array}\right.$$

where $G\left(\sigma \right)=\frac{1}{2}\left(\frac{1-\sigma^{T}\sigma }{2}I_{3}+S\left(\sigma \right)+\sigma \sigma^{T}\right)\in {\mathbb{R}}^{3\times 3}$. $\sigma \in {\mathbb{R}}^{3}$, $\omega \in {\mathbb{R}}^{3}$, $r\in {\mathbb{R}}^{3}$, and $v\in {\mathbb{R}}^{3}$ are the attitude, angular velocity, position, and velocity of the chaser with respect to the earth center in frame $F_{c}$. $u_{\tau }\in {\mathbb{R}}^{3}$ and $u_{f}\in {\mathbb{R}}^{3}$ are the control torques and forces produced by the actuators. $d_{\tau }\in {\mathbb{R}}^{3}$ and $d_{f}\in {\mathbb{R}}^{3}$ are the disturbance torques and forces acting on the chaser. $J\in {\mathbb{R}}^{3\times 3}$ and m∈ℝ denote the inertia matrix and mass of the chaser. The notation S(ω) stands for the skew-symmetric matrix of ω, denoted as

(2) $$S\left(\omega \right)=\left[\begin{array}{ccc} 0 & -\omega_{3} & \omega_{2}\\ \omega_{3} & 0 & -\omega_{1}\\ -\omega_{2} & \omega_{1} & 0 \end{array}\right].$$

where $\Gamma_{\tau }=\mathrm{diag}\left\{\gamma_{\tau 1},\gamma_{\tau 2},\gamma_{\tau 3}\right\}$ and $\Gamma_{f}=\mathrm{diag}\left\{\gamma_{f1},\gamma_{f2},\gamma_{f3}\right\}$ are the actuator health factor matrices, with $0\leq \gamma_{\tau i}\leq 1$ and $0\leq \gamma_{fi}\leq 1$ (i=1,2,3). The case $\gamma_{\tau i}=1$ and $\gamma_{fi}=1$ means the corresponding control torque and force are healthy. The case $0<\gamma_{\tau i}<1$ and $0<\gamma_{fi}<1$ means the corresponding control torque and force are partially faulty. The case $\gamma_{\tau i}=0$ and $\gamma_{fi}=0$ means the corresponding control torque and force are completely failed. In this paper, the chaser is assumed to be fully actuated with $0<\gamma_{\tau i}\leq 1$ and $0<\gamma_{fi}\leq 1$ (i=1,2,3).

Similarly, the attitude and position dynamics of the target can be expressed in frame $F_{t}$ as

(3) $$\left\{ \begin{array}{l} {\dot{\sigma }}_{t}=G\left(\sigma_{t}\right)\omega_{t},\\ J_{t}{\dot{\omega }}_{t}+S\left(\omega_{t}\right)J_{t}\omega_{t}=h_{\tau },\\ {\dot{r}}_{t}=v_{t}-S\left(\omega_{t}\right)r_{t},\\ m_{t}{\dot{v}}_{t}+m_{t}S\left(\omega_{t}\right)v_{t}=h_{f}, \end{array}\right.$$

where $\sigma_{t}\in {\mathbb{R}}^{3}$, $\omega_{t}\in {\mathbb{R}}^{3}$, $r_{t}\in {\mathbb{R}}^{3}$, and $v_{t}\in {\mathbb{R}}^{3}$ are the attitude, angular velocity, position, and velocity of the target with respect to the earth center in frame $F_{t}$. $h_{\tau }\in {\mathbb{R}}^{3}$ and $h_{f}\in {\mathbb{R}}^{3}$ are the disturbance torques and forces acted on the target. $J_{t}\in {\mathbb{R}}^{3\times 3}$ and $m_{t}\in \mathbb{R}$ denote the inertia matrix and mass of the target.

According to the geometric relationship in Figure 1, the position and velocity of the point P with respect to the earth center in frame $F_{t}$ can be expressed as

(4) $$\left\{ \begin{array}{l} r_{p}=r_{t}+p_{t},\\ v_{p}=v_{t}+S\left(\omega_{t}\right)p_{t}, \end{array}\right.$$

where $p_{t}$ is the constant position vector of the point P with respect to the target in frame $F_{t}$. The relative attitude, angular velocity, position, and velocity of the target with respect to the chaser can be defined in frame $F_{p}$ as

(5) $$\left\{ \begin{array}{l} \sigma_{e}=\sigma ⊗\sigma_{t}^{-1}=\frac{\left(1-\sigma_{t}^{T}\sigma_{t}\right)\sigma -\left(1-\sigma^{T}\sigma \right)\sigma_{t}-2S\left(\sigma_{t}\right)\sigma }{1+\sigma_{t}^{T}\sigma_{t}\sigma^{T}\sigma +2\sigma_{t}^{T}\sigma },\\ \omega_{e}=\omega -R\left(\sigma_{e}\right)\omega_{t},\\ r_{e}=r-R\left(\sigma_{e}\right)r_{p},\\ v_{e}=v-R\left(\sigma_{e}\right)v_{p}, \end{array}\right.$$

where $R\left(\sigma_{e}\right)=I_{3}+\frac{8S^{2}\left(\sigma_{e}\right)-4\left(1-\sigma_{e}^{T}\sigma_{e}\right)S\left(\sigma_{e}\right)}{{\left(1+\sigma_{e}^{T}\sigma_{e}\right)}^{2}}\in {\mathbb{R}}^{3\times 3}$ is the rotation matrix from frame $F_{t}$ to frame $F_{p}$. The matrix $R\left(\sigma_{e}\right)$ has the property $\dot{R}\left(\sigma_{e}\right)=-S\left(\omega_{e}\right)R\left(\sigma_{e}\right)$.

Note that ${\dot{r}}_{p}=v_{p}-S\left(\omega_{t}\right)r_{p}$ and ${\dot{\omega }}_{t}=-J_{t}^{-1}S\left(R^{T}\left(\omega -\omega_{e}\right)\right)J_{t}R^{T}\left(\omega -\omega_{e}\right)+J_{t}^{-1}\omega_{t}$. Substituting (1), (3), and (4) into (5), the relative attitude and position dynamics of the target with respect to the chaser can be obtained in frame $F_{p}$ as

(6) $${\dot{\sigma }}_{e}=G\left(\sigma_{e}\right)\omega_{e},$$

(7) $$J{\dot{\omega }}_{e}=\Gamma_{\tau }u_{\tau }+\zeta_{\tau },$$

(8) $${\dot{r}}_{e}=v_{e}-S\left(\omega \right)r_{e},$$

(9) $$m{\dot{v}}_{e}=\Gamma_{f}u_{f}+\zeta_{f},$$

where $\zeta_{\tau }$ and $\zeta_{f}$ are the lumped unknowns in the relative attitude and position dynamics, given as

(10) $$\zeta_{\tau }=-S\left(\omega \right)J\omega +S\left(\omega \right)J\omega_{e}-JR\left(\sigma_{e}\right)J_{t}^{-1}S\left(R^{T}\left(\sigma_{e}\right)\left(\omega -\omega_{e}\right)\right)J_{t}R^{T}\left(\sigma_{e}\right)\left(\omega -\omega_{e}\right)-d_{\tau }+JR\left(\sigma_{e}\right)J_{t}^{-1}h_{\tau },$$

(11) $$\begin{array}{l} \zeta_{f}=-mS\left(\omega \right)v_{e}-mS^{2}\left(\omega -\omega_{e}\right)R\left(\sigma_{e}\right)p_{t}-mR\left(\sigma_{e}\right)S\left(p_{t}\right)J_{t}^{-1}S\left(R^{T}\left(\sigma_{e}\right)\left(\omega -\omega_{e}\right)\right)J_{t}R^{T}\left(\sigma_{e}\right)\left(\omega -\omega_{e}\right)\\ \;\;\;\;\;\;\;\;+d_{f}-\frac{mR\left(\sigma_{e}\right)h_{f}}{m_{t}}+mR\left(\sigma_{e}\right)S\left(p_{t}\right)J_{t}^{-1}h_{\tau }. \end{array}$$

**Remark** **1.** *From the 6-DOF dynamic model of spacecraft proximity operations (8) and (9), the relative translational motion of the target with respect to the chaser is heavily affected by the relative rotational motion due to the inherent coupling between the relative attitude and position*.

### 2.2. Purpose

The purpose of this research is to design a controller for the spacecraft proximity operation system such that relative attitude $\sigma_{e}$, angular velocity $\omega_{e}$, position $r_{e}$, and velocity $v_{e}$ can stabilize into the small neighborhoods around the origin, even in the presence of unknown parameters, disturbances, and actuator faults.

### 2.3. Neural Approximation

**Lemma** **1.** Ref. [31] *For any continuous nonlinear function* f(Z)*,* $Z\in {\mathbb{R}}^{n}$*, it can be approximated by a radial basis function NN (RBFNN) as*

(12) $$f\left(Z\right)=W^{*T}\Phi \left(Z\right)+\varepsilon \left(Z\right),$$

*where* $W^{*}\in {\mathbb{R}}^{N}$ *is the ideal RBFNN weight,* $\Phi \left(Z\right)={\left[\phi_{1}\left(Z\right),\phi_{2}\left(Z\right),\ldots ,\phi_{N}\left(Z\right)\right]}^{T}$ *is the basis function vector,* ε(Z) *is the identification error satisfying* $\left|\varepsilon \left(Z\right)\right|\leq \bar{\varepsilon }$ *,* $\bar{\varepsilon }$ *is a positive constant, and* N *is the number of RBFNN nodes. Moreover,* $\varphi_{i}\left(Z\right)$ *is commonly chosen as the Gaussian function*

(13) $$\varphi_{i}\left(Z\right)=\exp\left(-{\left\|Z-c_{i}\right\|}^{2}/w_{i}^{2}\right),\;\;i=1,2,\ldots ,N,$$

*where* $c_{i}={\left[c_{i1},c_{i2},\ldots ,c_{in}\right]}^{T}\in {\mathbb{R}}^{n}$ *, and* $w_{i}$ *are the center and width of the Gaussian function, respectively.*

## 3. Control Design Methodology

### 3.1. Architecture of the Whole Control Design

**Figure 2:**
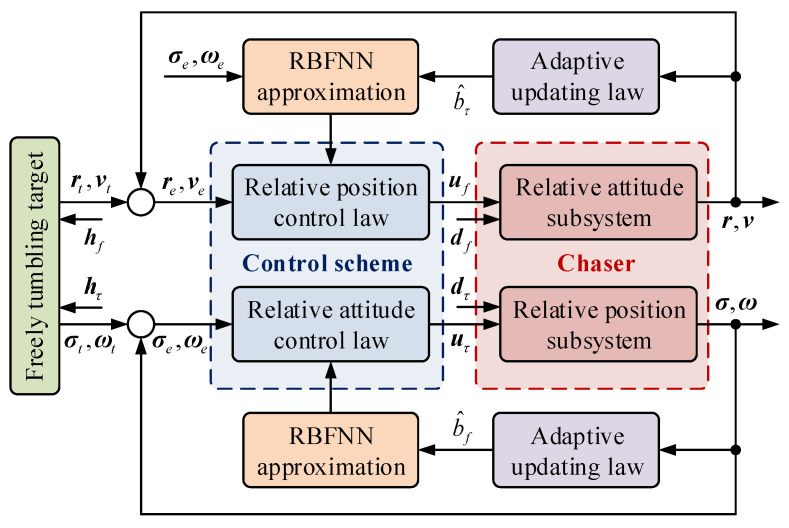
Architecture of the whole control design.

The structure of the proposed neural adaptive fault-tolerant control scheme is shown in Figure 2. Specifically, the proposed controller is made up of a relative position control law and a relative attitude control law. Both the relative position control law and relative attitude control law are designed by adopting the NNs to approximate the upper bound of the lumped unknowns. The ultimate uniform boundedness of the overall closed-loop system is achieved through Lyapunov analysis.

### 3.2. Relative Attitude Control Design

First, consider the relative attitude subsystem described as (6) and (7). Introduce the following filtered error:

(14) $$s_{1}=\omega_{e}+\alpha_{1}\sigma_{e},$$

where $\alpha_{1}>0$. Evaluating the time differentiation of $s_{1}$ yields

(15) $$J{\dot{s}}_{1}=\Gamma_{\tau }u_{\tau }+\xi_{\tau },$$

where $\xi_{\tau }=\zeta_{\tau }+\alpha_{1}G\left(\sigma_{e}\right)\omega_{e}$. Define the input variable $Z_{\tau }={\left[\sigma_{e}^{T},\omega_{e}^{T}\right]}^{T}$. By Lemma 1, the lumped uncertainty can be expressed as

(16) $$\xi_{\tau }=W_{\tau }^{*T}\Phi_{\tau }\left(Z_{\tau }\right)+\varepsilon_{\tau }\left(Z_{\tau }\right),$$

where $W_{\tau }^{*}\in {\mathbb{R}}^{N\times 3}$ is the ideal RBFNN weight, $\Phi_{\tau }\left(Z_{\tau }\right)\in {\mathbb{R}}^{N}$ is the Gaussian basis function vector, $\varepsilon_{\tau }\left(Z_{\tau }\right)\in {\mathbb{R}}^{3}$ is the approximation error satisfying $\left\|\varepsilon_{\tau }\left(Z_{\tau }\right)\right\|\leq {\bar{\varepsilon }}_{\tau }$, ${\bar{\varepsilon }}_{\tau }$ is a positive constant, and N is the number of RBFNN nodes. Note that $\left\|W_{\tau }^{*}\right\|\leq {\bar{W}}_{\tau }$. Substituting it into (16) yields

(17) $$\begin{array}{l} \left\|\xi_{\tau }\right\|\leq \left\|W_{\tau }^{*}\right\|\left\|\Phi_{\tau }\left(Z_{\tau }\right)\right\|+\left\|\varepsilon_{\tau }\left(Z_{\tau }\right)\right\|\\ \;\;\;\;\;\;\leq b_{\tau }\Phi_{\tau }, \end{array}$$

where $b_{\tau }=\max\left\{{\bar{W}}_{\tau },{\bar{\varepsilon }}_{\tau }\right\}$ is an unknown constant, and $\Phi_{\tau }=\left\|\Phi_{\tau }\left(Z_{\tau }\right)\right\|+1$ is a known function. Then, the relative attitude control law is designed as

(18) $$u_{\tau }=-k_{1}s_{1}-\eta_{1}{\hat{b}}_{\tau }\Phi_{\tau }^{2}s_{1},$$

where $k_{1}>0$, $\eta_{1}>0$, and ${\hat{b}}_{\tau }$ is the estimation of $b_{\tau }$. Moreover, the adaptive updating law is designed as

(19) $${\dot{\hat{b}}}_{\tau }=-\mu_{1}{\hat{b}}_{\tau }+\eta_{1}\Phi_{\tau }^{2}{\left\|s_{\tau }\right\|}^{2},$$

where $\mu_{1}>0$.

**Theorem** **1.** *When the relative attitude control law (18) and the adaptive updating law (19) are employed to the relative attitude subsystem described as (6) and (7), the overall closed-loop system is ultimately uniformly bounded and the relative attitude* $\sigma_{e}$, and angular velocity $\omega_{e}$*can stabilize into the small neighborhoods around the origin*.

**Proof.**  Introduce the following Lyapunov function:

(20) $$V_{1}=\frac{1}{2}s_{1}^{T}Js_{1}+\frac{1}{2\gamma_{\tau \min}}{\tilde{b}}_{\tau }^{2},$$

where $\gamma_{\tau \min}=\min\left\{\gamma_{\tau 1},\gamma_{\tau 2},\gamma_{\tau 3}\right\}$, and ${\tilde{b}}_{\tau }=b_{\tau }-\gamma_{\tau \min}{\hat{b}}_{\tau }$ denotes the estimation error of $b_{\tau }$. Evaluating the time differentiation of $V_{1}$ yields

(21) $$\begin{array}{l} {\dot{V}}_{1}=s_{1}^{T}J{\dot{s}}_{1}-{\tilde{b}}_{\tau }{\dot{\hat{b}}}_{\tau }\\ \;\;\;\;=s_{1}^{T}\left(\Gamma_{\tau }u_{\tau }+\xi_{\tau }\right)-{\tilde{b}}_{\tau }{\dot{\hat{b}}}_{\tau }. \end{array}$$

Substituting the relative attitude control law (18) and the adaptive updating law (19), we have

(22) $$\begin{array}{l} {\dot{V}}_{1}=s_{1}^{T}\left(\Gamma_{\tau }\left(-k_{1}s_{1}-\eta_{1}{\hat{b}}_{\tau }\Phi_{\tau }^{2}s_{1}\right)+\xi_{\tau }\right)-{\tilde{b}}_{\tau }\left(-\mu_{1}{\hat{b}}_{\tau }+\eta_{1}\Phi_{\tau }^{2}{\left\|s_{\tau }\right\|}^{2}\right)\\ \;\;\;\;=-\gamma_{\tau \min}k_{1}{\left\|s_{1}\right\|}^{2}-\eta_{1}b_{\tau }\Phi_{\tau }^{2}{\left\|s_{1}\right\|}^{2}+s_{1}^{T}\xi_{\tau }+\mu_{1}{\tilde{b}}_{\tau }{\hat{b}}_{\tau }. \end{array}$$

Consider the following inequalities:

(23) $$s_{1}^{T}\xi_{\tau }\leq b_{\tau }\Phi_{\tau }{\left\|s_{1}\right\|}^{2}\leq \eta_{1}b_{\tau }\Phi_{\tau }^{2}{\left\|s_{1}\right\|}^{2}+\frac{1}{4\eta_{1}},$$

(24) $$\mu_{1}{\tilde{b}}_{\tau }{\hat{b}}_{\tau }=\frac{\mu_{1}}{\gamma_{\tau \min}}{\tilde{b}}_{\tau }\left(b_{\tau }-{\tilde{b}}_{\tau }\right)\leq \frac{\mu_{1}}{2\gamma_{\tau \min}}\left(b_{\tau }^{2}-{\tilde{b}}_{\tau }^{2}\right).$$

Substituting (23) and (24) into (22) yields

(25) $$\begin{array}{l} {\dot{V}}_{1}\leq -\gamma_{\tau \min}k_{1}{\left\|s_{1}\right\|}^{2}-\frac{\mu_{1}}{2\gamma_{\tau \min}}{\tilde{b}}_{\tau }^{2}+\frac{1}{4\eta_{1}}+\frac{\mu_{1}}{2\gamma_{\tau \min}}b_{\tau }^{2}\\ \;\;\;\;\leq -\kappa_{1}V_{1}+\vartheta_{1}, \end{array}$$

where $\kappa_{1}=\min\left\{\frac{2\gamma_{\tau \min}k_{1}}{\lambda_{\max}\left(J\right)},\mu_{1}\right\}$, and $\vartheta_{1}=\frac{1}{4\eta_{1}}+\frac{\mu_{1}}{2\gamma_{\tau \min}}b_{\tau }^{2}$. Solving inequality (25), we further have

(26) $$V_{1}\leq \left(V_{1}\left(0\right)-\frac{\vartheta_{1}}{\kappa_{1}}\right)e^{-\kappa_{1}t}+\frac{\vartheta_{1}}{\kappa_{1}}.$$

Combining with the definition of $V_{1}$, it follows that the overall closed-loop system is ultimately uniformly bounded, and the error signals $s_{1}$ and ${\tilde{b}}_{\tau }$ can stabilize into the small neighborhoods around the origin. Considering the definition of $s_{1}$, this further implies that the relative attitude $\sigma_{e}$ and angular velocity $\omega_{e}$ can stabilize into the small neighborhoods around the origin. The proof of Theorem 1 is thus finished. □

### 3.3. Relative Position Control Design

Then, consider the relative position subsystem described as (8) and (9). Introduce the following filtered error:

(27) $$s_{2}=v_{e}+\alpha_{2}r_{e},$$

where $\alpha_{2}>0$. Evaluating the time differentiation of $s_{2}$ yields

(28) $$m{\dot{s}}_{2}=\Gamma_{f}u_{f}+\xi_{f},$$

where $\xi_{\tau }=\zeta_{\tau }+\alpha_{2}\left(v_{e}-S\left(\omega \right)r_{e}\right)$. Define the input variable $Z_{f}={\left[\sigma_{e}^{T},\omega_{e}^{T}p_{e}^{T},v_{e}^{T}\right]}^{T}$. By Lemma 1, the lumped uncertainty can be expressed as

(29) $$\xi_{f}=W_{f}^{*T}\Phi_{f}\left(Z_{f}\right)+\varepsilon_{f}\left(Z_{f}\right),$$

where $W_{f}^{*}\in {\mathbb{R}}^{N\times 3}$ is the ideal RBFNN weight, $\Phi_{f}\left(Z_{f}\right)\in {\mathbb{R}}^{N}$ is the Gaussian basis function vector, $\varepsilon_{f}\left(Z_{f}\right)\in {\mathbb{R}}^{3}$ is the approximation error satisfying $\left\|\varepsilon_{f}\left(Z_{f}\right)\right\|\leq {\bar{\varepsilon }}_{f}$, ${\bar{\varepsilon }}_{f}$ is a positive constant, and N is the number of RBFNN nodes. Note that $\left\|W_{f}^{*}\right\|\leq {\bar{W}}_{f}$. Substituting it into (29) yields

(30) $$\begin{array}{l} \left\|\xi_{f}\right\|\leq \left\|W_{f}^{*}\right\|\left\|\Phi_{f}\left(Z_{f}\right)\right\|+\left\|\varepsilon_{f}\left(Z_{f}\right)\right\|\\ \;\;\;\;\;\;\leq b_{f}\Phi_{f}, \end{array}$$

where $b_{f}=\max\left\{{\bar{W}}_{f},{\bar{\varepsilon }}_{f}\right\}$ is an unknown constant, and $\Phi_{f}=\left\|\Phi_{f}\left(Z_{f}\right)\right\|+1$ is a known function. Then, the relative position control law is designed as

(31) $$u_{f}=-k_{2}s_{2}-\eta_{2}{\hat{b}}_{f}\Phi_{f}^{2}s_{2},$$

where $k_{2}>0$, $\eta_{2}>0$, and ${\hat{b}}_{f}$ is the estimation of $b_{f}$. Moreover, the adaptive updating law is designed as

(32) $${\dot{\hat{b}}}_{f}=-\mu_{2}{\hat{b}}_{f}+\eta_{2}\Phi_{f}^{2}{\left\|s_{f}\right\|}^{2},$$

where $\mu_{2}>0$.

**Theorem** **2.** *When the relative position control law (31) and the adaptive updating law (32) are employed to the relative position subsystem described as (8) and (9), the overall closed-loop system is ultimately uniformly bounded and the relative position*$r_{e}$, and velocity $v_{e}$*can stabilize into the small neighborhoods around the origin.*

**Proof.**  Introduce the following Lyapunov function:

(33) $$V_{2}=\frac{1}{2}ms_{2}^{T}s_{2}+\frac{1}{2\gamma_{f\min}}{\tilde{b}}_{f}^{2},$$

where $\gamma_{f\min}=\min\left\{\gamma_{f1},\gamma_{f2},\gamma_{f3}\right\}$, and ${\tilde{b}}_{f}=b_{f}-\gamma_{f\min}{\hat{b}}_{f}$ denotes the estimation error of $b_{f}$. Evaluating the time differentiation of $V_{2}$ yields

(34) $$\begin{array}{l} {\dot{V}}_{2}=ms_{2}^{T}{\dot{s}}_{2}-{\tilde{b}}_{f}{\dot{\hat{b}}}_{f}\\ \;\;\;\;=s_{2}^{T}\left(\Gamma_{f}u_{f}+\xi_{f}\right)-{\tilde{b}}_{f}{\dot{\hat{b}}}_{f}. \end{array}$$

Substituting the relative position control law (31) and the adaptive updating law (32), we have

(35) $$\begin{array}{l} {\dot{V}}_{2}=s_{2}^{T}\left(\Gamma_{f}\left(-k_{2}s_{2}-\eta_{2}{\hat{b}}_{f}\Phi_{f}^{2}s_{2}\right)+\xi_{f}\right)-{\tilde{b}}_{f}\left(-\mu_{2}{\hat{b}}_{f}+\eta_{2}\Phi_{f}^{2}{\left\|s_{f}\right\|}^{2}\right)\\ \;\;\;\;=-\gamma_{f\min}k_{2}{\left\|s_{2}\right\|}^{2}-\eta_{2}b_{f}\Phi_{f}^{2}{\left\|s_{2}\right\|}^{2}+s_{2}^{T}\xi_{f}+\mu_{2}{\tilde{b}}_{f}{\hat{b}}_{f}. \end{array}$$

Consider the following inequalities:

(36) $$s_{2}^{T}\xi_{f}\leq b_{f}\Phi_{f}{\left\|s_{2}\right\|}^{2}\leq \eta_{2}b_{f}\Phi_{f}^{2}{\left\|s_{2}\right\|}^{2}+\frac{1}{4\eta_{2}},$$

(37) $$\mu_{2}{\tilde{b}}_{f}{\hat{b}}_{f}=\frac{\mu_{2}}{\gamma_{f\min}}{\tilde{b}}_{f}\left(b_{f}-{\tilde{b}}_{f}\right)\leq \frac{\mu_{2}}{2\gamma_{f\min}}\left(b_{f}^{2}-{\tilde{b}}_{f}^{2}\right).$$

Substituting (36) and (37) into (35) yields

(38) $$\begin{array}{l} {\dot{V}}_{2}\leq -\gamma_{f\min}k_{2}{\left\|s_{2}\right\|}^{2}-\frac{\mu_{2}}{2\gamma_{f\min}}{\tilde{b}}_{f}^{2}+\frac{1}{4\eta_{2}}+\frac{\mu_{2}}{2\gamma_{f\min}}b_{f}^{2}\\ \;\;\;\;\leq -\kappa_{2}V_{2}+\vartheta_{2}, \end{array}$$

where $\kappa_{2}=\min\left\{\frac{2\gamma_{f\min}k_{2}}{m},\mu_{2}\right\}$ and $\vartheta_{2}=\frac{1}{4\eta_{2}}+\frac{\mu_{2}}{2\gamma_{f\min}}b_{f}^{2}$. Solving inequality (38), we further have

(39) $$V_{2}\leq \left(V_{2}\left(0\right)-\frac{\vartheta_{2}}{\kappa_{2}}\right)e^{-\kappa_{2}t}+\frac{\vartheta_{2}}{\kappa_{2}}.$$

Combined with the definition of $V_{2}$, it follows that the overall closed-loop system is ultimately uniformly bounded, and error signals $s_{2}$ and ${\tilde{b}}_{f}$ can stabilize into the small neighborhoods around the origin. Considering the definition of $s_{2}$, this further implies that the relative position $r_{e}$ and velocity $v_{e}$ can stabilize into the small neighborhoods around the origin. The proof of Theorem 2 is thus finished. □

**Remark** **2.** *In the conventional intelligent approximation [28,29,30], the NNs and fuzzy logic systems are introduced to directly approximate the lumped unknowns, and the number of the adaptive parameters is* 2×3N. *Alternatively, the indirect neural approximation is exploited in this paper by adopting NNs to approximate the upper bound of the lumped unknowns. In this way, only two adaptive parameters,*${\hat{b}}_{\tau }$ and ${\hat{b}}_{f}$*, are required for the* *indirect neural approximation, and the online calculation burden of the proposed controller is therefore significantly reduced. Actually, the* *indirect neural approximation makes the proposed controller more suitable for practical engineering, especially considering the onboard computer has limited online calculation capability*.

**Remark** **3.** *According to Theorems 1 and 2, the proposed controller can ensure the relative attitude, angular velocity, position, and velocity stabilize into the small neighborhoods around the origin. From (26) and (39), it follows that the small neighborhoods around the origin are adjustable. If we set the parameters* $\alpha_{1}$, $\alpha_{2}$, $k_{1}$, and $k_{2}$*as large as required, the small neighborhoods can be made sufficiently small.*

**Remark** **4.** *It is noteworthy that the RBFNN utilized for intelligent control in this paper can also be replaced by some other approximation tools, such as recurrent NNs, wavelet NNs, and fuzzy logic systems. Moreover,adaptive dynamic programming is an effective methodology for the optimal control of unknown nonlinear systems with the help of critic NNs [32,33,34,35]. Future investigation building on this research will focus on extending the present results by embedding them with an adaptive dynamic programming approach*.

## 4. Simulated Example

A simulated example is performed to illustrate the proposed control scheme. The sampling frequency for feedback is set as $f_{s}=20\;\mathrm{Hz}$. The initial attitude, angular velocity, position, and velocity of the chaser are given as $\sigma \left(0\right)={\left[0,0,0\right]}^{T}$, $\omega \left(0\right)={\left[0,0,0\right]}^{T}\;\mathrm{rad}/s$, $r\left(0\right)={\left[1,1,1\right]}^{T}\times 7.078\times 10^{6}\;m$, and $v\left(0\right)={\left[2,3,-2\right]}^{T}\;m/s$. The initial relative attitude, angular velocity, position, and velocity of the target with respect to the chaser are given as $\sigma_{e}\left(0\right)={\left[0.2,-0.4,0.3\right]}^{T}$, $\omega_{e}\left(0\right)={\left[0.02,-0.02,0.02\right]}^{T}\;\mathrm{rad}/s$, $r_{e}\left(0\right)={\left[50\sqrt{2},0,-50\sqrt{2}\right]}^{T}\;m$, and $v_{e}\left(0\right)={\left[0.5,-0.5,0.5\right]}^{T}\;m/s$. Moreover, the constant position vector of the desired docking point with respect to the target in frame $F_{t}$ is given as $p_{t}={\left[0,5,0\right]}^{T}\;m$. The inertia matrices of the chaser and the target are chosen as

(40) $$J=\left[\begin{array}{ccc} 38 & -2.5 & -5.5\\ -2.5 & 44 & -2.7\\ -5.5 & -2.7 & 36 \end{array}\right]\;\mathrm{kg}\cdot m^{2},$$

(41) $$J_{t}=\left[\begin{array}{ccc} 3336 & -135.4 & -154.2\\ -135.4 & 3184 & -148.5\\ -154.2 & -148.5 & 2423 \end{array}\right]\;\mathrm{kg}\cdot m^{2}.$$

The masses of the chaser and the target are chosen as m=58.2 kg, and $m_{t}=5425.6\;\mathrm{kg}$. The inertia matrices and the masses are assumed to be fully unknown for the control design. The disturbance torques and forces acted on the chaser and the target are chosen as

(42) $$d_{\tau }=h_{\tau }=\left[\begin{array}{c} 1+\sin\left(\pi t/125\right)+\sin\left(\pi t/200\right)\\ 1+\sin\left(\pi t/125\right)+\sin\left(\pi t/250\right)\\ 1+\cos\left(\pi t/125\right)+\cos\left(\pi t/250\right) \end{array}\right]\times 10^{-5}\;\mathrm{Nm},$$

(43) $$d_{f}=h_{f}=\left[\begin{array}{c} 1+\sin\left(\pi t/125\right)+\sin\left(\pi t/200\right)\\ 1+\sin\left(\pi t/125\right)+\sin\left(\pi t/250\right)\\ 1+\cos\left(\pi t/125\right)+\cos\left(\pi t/250\right) \end{array}\right]\times 10^{-4}\;N.$$

Due to the actuator saturation, the acceptable maximum control torques and forces are set as

(44) $$\left|u_{\tau i}\right|\leq 2\;\mathrm{Nm},\;\;\left|u_{fi}\right|\leq 200\;N,\;\;i=1,2,3.$$

In addition, the actuator faults are also considered. The actuator health factor matrices are given as

(45) $$\Gamma_{\tau }=\mathrm{diag}\left\{0.8+0.1\sin(0.1t),0.8-0.1\cos(0.3t),0.7-0.2\sin(0.2t)\right\},$$

(46) $$\Gamma_{f}=\mathrm{diag}\left\{0.7+0.1\sin(0.2t),0.6+0.2\cos(0.1t),0.8+0.2\cos(0.1t)\right\}.$$

The commonly used proportional-derivative (PD) controller is employed for performance comparisons. The compared PD controller is also made up of a relative attitude control law and a relative position control law. The relative attitude control law is designed as

(47) $$u_{\tau }=-k_{p1}\sigma_{e}-k_{d1}\omega_{e},$$

where $k_{p1}>0$ and $k_{d1}>0$. Moreover, the relative position control law is designed as

(48) $$u_{f}=-k_{p2}r_{e}-k_{d2}v_{e},$$

where $k_{p2}>0$ and $k_{d2}>0$.

The parameters of the proposed neural adaptive fault-tolerant controller are given as $\alpha_{1}=0.5$, $\alpha_{2}=0.5$, $k_{1}=20$, $k_{2}=20$, $\mu_{1}=1$, $\mu_{2}=1$, $\eta_{1}=0.1$, and $\eta_{2}=0.1$. Seven nodes are selected for the hidden layer of the RBFNN. The parameters of the RBFNN are selected as $c_{i}={\left[-3,-2,-1,0,1,2,3\right]}^{T}$, and $w_{i}=6$. The initial values of the adaptive parameters are set as ${\hat{b}}_{\tau }=0$ and ${\hat{b}}_{f}=0$. Additionally, the parameters of the compared PD controller are given as $k_{p1}=12$, $k_{p2}=16$, $k_{d1}=12$, and $k_{d2}=16$.

**Figure 3:**
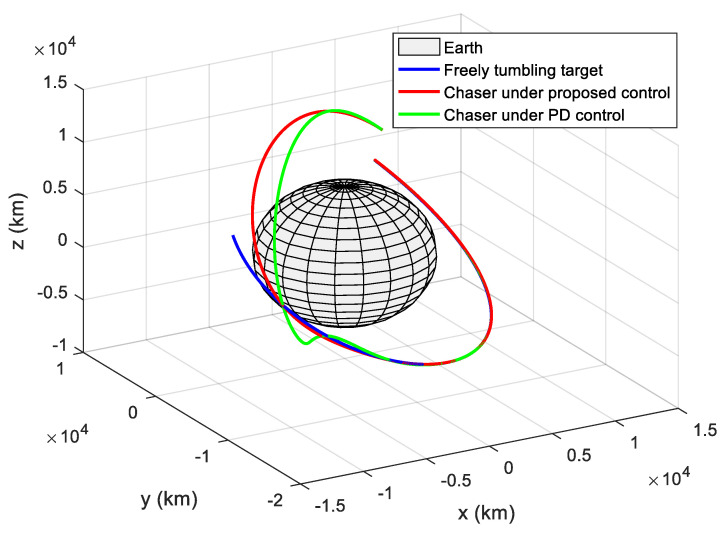
Translational motion of the chaser and the target for proximity operation.

**Figure 4:**
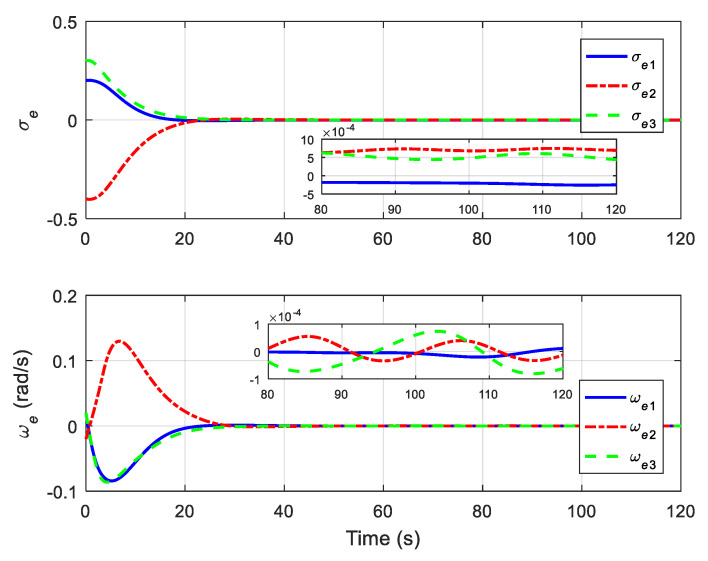
Relative attitude and angular velocity under the proposed controller.

**Figure 5:**
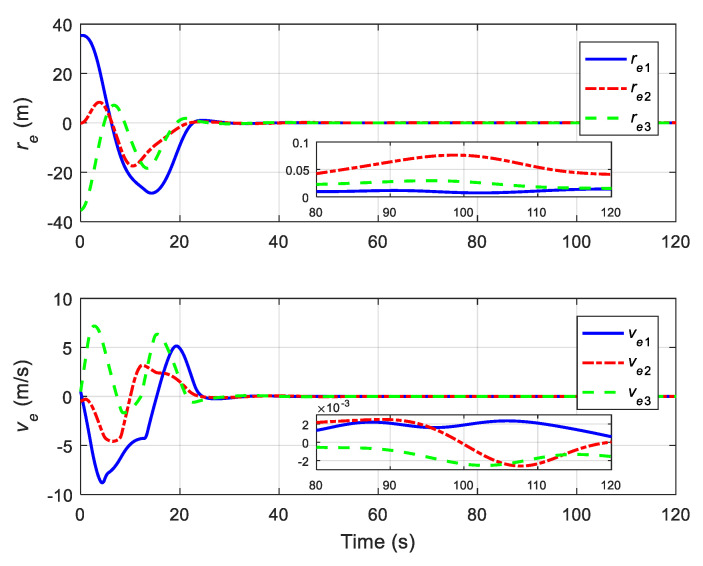
Relative position and velocity under the proposed controller.

**Figure 6:**
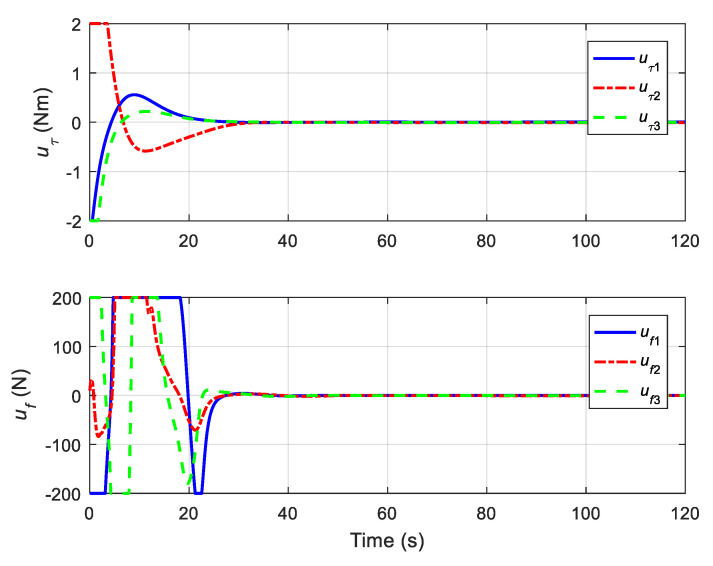
Control torques and forces of the chaser under the proposed controller.

**Figure 7:**
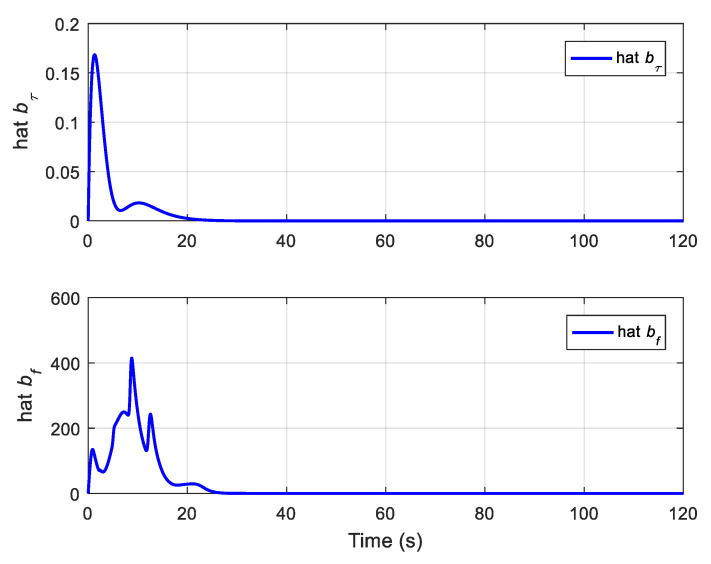
Two adaptive parameters under the proposed controller.

**Figure 8:**
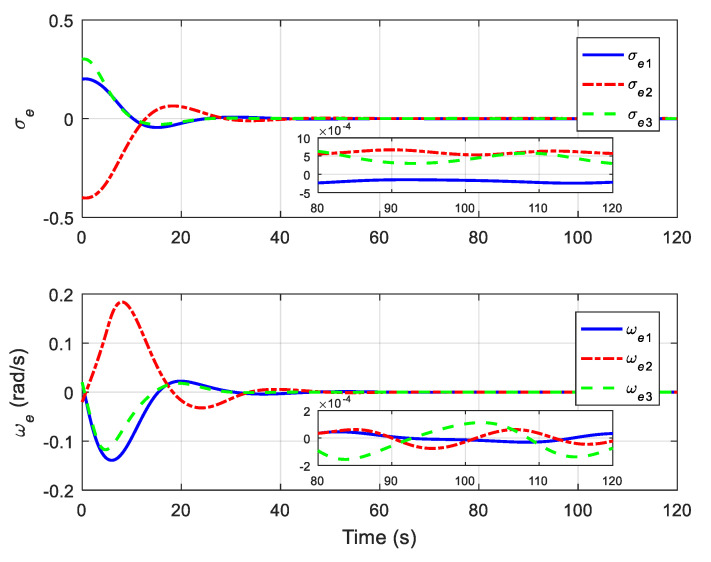
Relative attitude and angular velocity under the PD controller.

**Figure 9:**
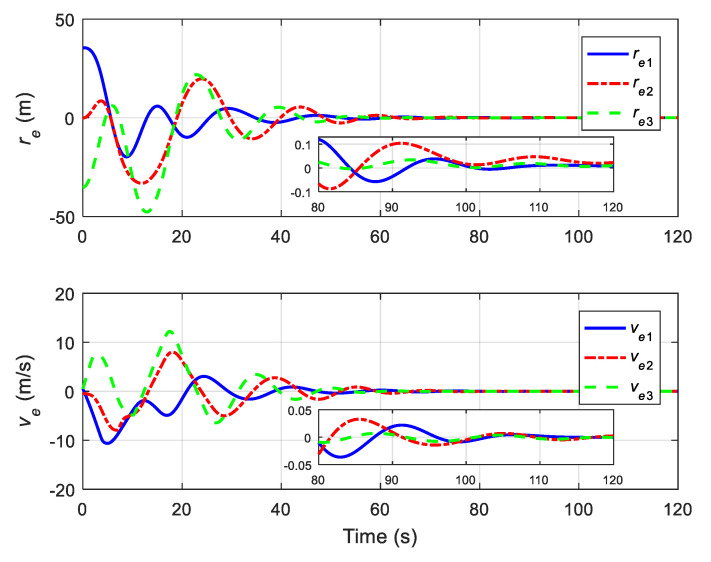
Relative position and velocity under the PD controller.

**Figure 10:**
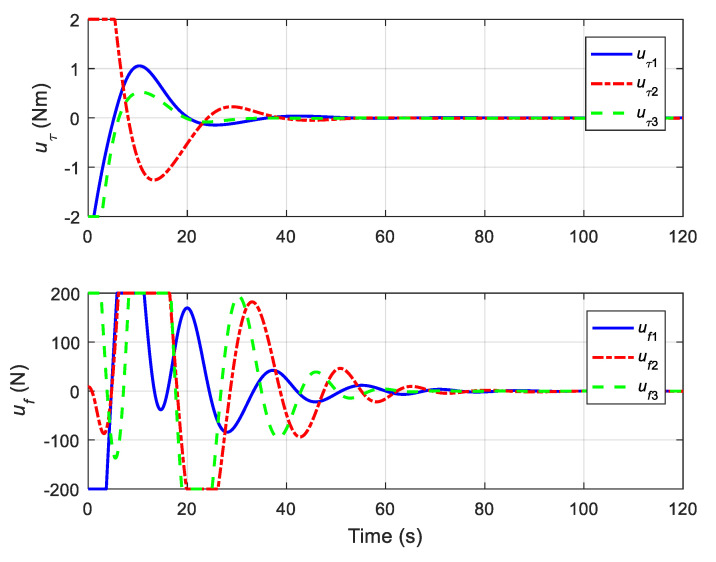
Control torques and forces of the chaser under the PD controller.

The translational motion of the chaser and the target for proximity operation is provided in Figure 3. It is clearly seen that the chaser, under both the proposed neural adaptive fault-tolerant controller and the compared PD controller, can quickly approach the target, and the spacecraft proximity operation can be well accomplished. Specifically, the simulation results of the proposed neural adaptive fault-tolerant controller are given in Figure 4, Figure 5, Figure 6 and Figure 7. Figure 4 shows the time profiles of the relative attitude and angular velocity under the proposed controller. The time profiles of the relative position and velocity under the proposed controller are presented in Figure 5. Figure 6 gives the time profiles of the control torques and forces of the chaser under the proposed controller. The changing curves of two adaptive parameters under the proposed controller are depicted in Figure 7. Moreover, the simulation results of the compared PD controller are given in Figure 8, Figure 9 and Figure 10.

From Figure 4, Figure 5, Figure 8 and Figure 9, it is revealed that the steady-state relative errors under the PD controller are much larger than those under the proposed controller. Meanwhile, the PD controller has the obvious unexpected overshooting problem, which the proposed controller does not have. The proposed controller can achieve superior performance, even in the presence of unknown parameters, disturbances, and actuator faults. Nevertheless, the performance of the PD controller is relatively poor, due to the existence of lumped unknowns in 6-DOF dynamics. Benefiting from the indirect neural approximation, the proposed controller is robust against unknown parameters and disturbances and is also insensitive to actuator faults. Figure 6 and Figure 10 reveal that the control torques and forces of the chaser can always satisfy the actuator saturation constraints during the spacecraft proximity operation. From Figure 7, it can be inferred that the two adaptive parameters are bounded and change with time smoothly.

Furthermore, some crucial indexes are introduced to quantitatively compare the performance between the proposed controller and the PD controller. Specifically, the integrated absolute errors (IAEs) are defined as $IAE_{\sigma }=\sum_{i=1}^{3}\int_{0}^{t}\left|\sigma_{ei}\left(\tau_{s}\right)\right|d\tau_{s}$, $IAE_{\omega }=\sum_{i=1}^{3}\int_{0}^{t}\left|\omega_{ei}\left(\tau_{s}\right)\right|d\tau_{s}$, $IAE_{r}=\sum_{i=1}^{3}\int_{0}^{t}\left|r_{ei}\left(\tau_{s}\right)\right|d\tau_{s}$, and $IAE_{v}=\sum_{i=1}^{3}\int_{0}^{t}\left|v_{ei}\left(\tau_{s}\right)\right|d\tau_{s}$, which evaluates the steady-state response performance of the controller. Moreover, the integrated time absolute errors (ITAEs) are defined as $ITAE_{\sigma }=\sum_{i=1}^{3}\int_{0}^{t}\tau_{s}\left|\sigma_{ei}\left(\tau_{s}\right)\right|d\tau_{s}$, $ITAE_{\omega }=\sum_{i=1}^{3}\int_{0}^{t}\tau_{s}\left|\omega_{ei}\left(\tau_{s}\right)\right|d\tau_{s}$, $ITAE_{r}=\sum_{i=1}^{3}\int_{0}^{t}\tau_{s}\left|r_{ei}\left(\tau_{s}\right)\right|d\tau_{s}$, and $ITAE_{v}=\sum_{i=1}^{3}\int_{0}^{t}\tau_{s}\left|v_{ei}\left(\tau_{s}\right)\right|d\tau_{s}$, which evaluates the transient response performance of the controller. The total time for performance comparison is set as t=120 s. The IAEs and ITAEs under the proposed controller are $IAE_{\sigma }=3.64$, $IAE_{\omega }=1.70$, $IAE_{r}=424.95$, $IAE_{v}=116.73$, $ITAE_{\sigma }=28.16$, $ITAE_{\omega }=15.83$, $ITAE_{r}=4480.8$, and $ITAE_{v}=1265.6$. By contrast, the IAEs and ITAEs under the PD controller are $IAE_{\sigma }=3.75$, $IAE_{\omega }=2.33$, $IAE_{r}=797.86$, $IAE_{v}=243.22$, $ITAE_{\sigma }=30.55$, $ITAE_{\omega }=25.12$, $ITAE_{r}=13900$, and $ITAE_{v}=4695.2$. It is not difficult to find that the IAEs and ITAEs under the PD controller are much larger than those under the proposed controller. This means that the proposed controller can achieve better steady-state and transient responses than the PD controller.

In summary, the simulation results indicate that the proposed neural adaptive fault-tolerant controller can realize superior performance and good uncertainty rejection capability, which guarantees the successful implementation of the spacecraft proximity operation.

## 5. Conclusions

This paper aimed to propose a neural, adaptive, fault-tolerant control scheme for the integrated attitude and position control of spacecraft proximity operations in the presence of unknown parameters, disturbances, and actuator faults. The proposed controller is made up of a relative attitude control law and a relative position control law. Both the relative attitude control law and relative position control law were designed by adopting the NNs to approximate the upper bound of the lumped unknowns. By introducing the indirect neural approximation, the proposed controller is more suitable for practical engineering, especially considering the onboard computer has limited online calculation capability. The ultimate uniform boundedness of the overall closed-loop system can be achieved through Lyapunov analysis. The proposed controller can ensure the relative attitude, angular velocity, position, and velocity stabilize into the small neighborhoods around the origin. Lastly, simulation results indicate the effectiveness and superior performance of the proposed control scheme.

## Data Availability

Not applicable.
